# Supplementary figures and images for: Gender specific differences in COVID-19 knowledge, behavior and health effects among adolescents and young adults in Uttar Pradesh and Bihar, India
Source: PLoS One. 2020 Dec 17;15(12):e0244053. doi: 10.1371/journal.pone.0244053 (PMC7746145; doi:10.1371/journal.pone.0244053)

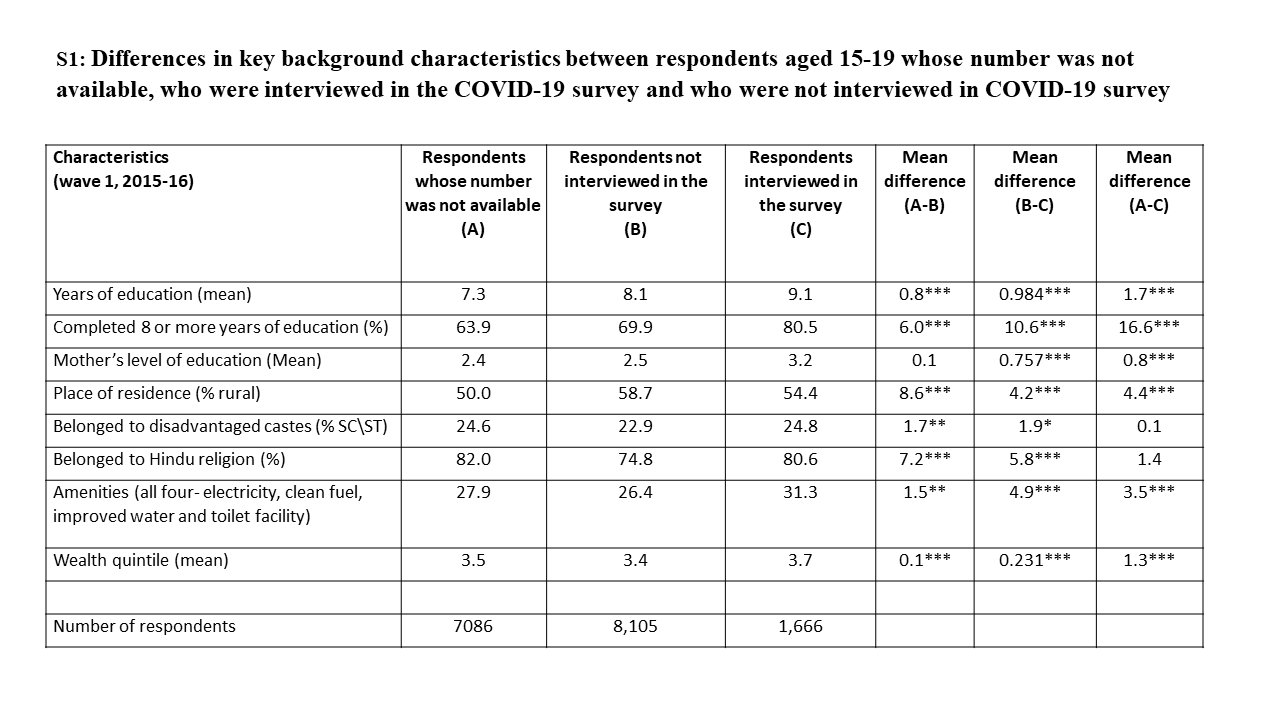

Supplement: S1 Table — (TIF) [file pone.0244053.s001.tif]
