# Supplementary material for: Gender specific differences in COVID-19 knowledge, behavior and health effects among adolescents and young adults in Uttar Pradesh and Bihar, India
Source: PLoS One. 2020 Dec 17;15(12):e0244053. doi: 10.1371/journal.pone.0244053 (PMC7746145; doi:10.1371/journal.pone.0244053)
Supplement: S1 File — (PDF) [file pone.0244053.s002.pdf]

POPULATION COUNCIL
Ideas. Evidence. Impact.

COVID-19-related knowledge, attitudes, and practices among adolescents and young people in Bihar and Uttar Pradesh, India

Authors: Rajib Acharya[1], Mukta Gundi[1], Thoai D. Ngo[2], Neelanjana Pandey[1], Sangram K. Patel[1], Jessie Pinchoff[2], Shilpi Rampal[1], Niranjan Saggurti[1,3], K.G. Santhya[1,3], Corinne White[2], A.J.F. Zavier[1]

Affiliations:

1. Population Council, New Delhi, India
2. Poverty, Gender, and Youth Program, Population Council, New York, NY
3. Population Council Institute, New Delhi, India

Corresponding authors:
K.G. Santhya, PhD
Senior Associate, India, Poverty, Gender, and Youth Program
+91 11 2464 2901
kgsanthya@popcouncil.org

Thoai D. Ngo, PhD, MHS
Director, Poverty, Gender, and Youth Program
(212) 339-0500
tngo@popcouncil.org

COVID-19 survey with UDAYA cohort

IDENTIFICATION	
STATE ________________________________	☐☐
PSU NUMBER ………………………………………………………...	☐☐☐
HOUSEHOLD NUMBER ……………………………………………..	☐☐☐
LINE NUMBER OF RESPONDENT ……………………………….…	☐
URBAN / RURAL ……………URBAN = 1, RURAL = 2	☐
MOBILE NUMBER	☐☐☐☐☐☐☐☐☐☐

INTERVIEWER VISITS		
	1	FINAL VISIT
DATE	_______	DAY ☐☐
		MONTH ☐☐
INTERVIEWER'S NAME	_______	YEAR ☐☐☐☐
		NAME CODE ☐☐☐
RESULT*	_______	RESULT CODE ☐
TIME SPENT	_______	TOTAL TIME ☐ HR. ☐☐ MIN

*RESULT CODES:
1. COMPLETED
2. PHONE OUT OF SERVICE /NO INCOMING /ETC
3. REFUSED /NOT RESPONDING DESPITE 3 CALLS /PHONE ENGAGED
4. PARENT/GUARDIAN REFUSED
8. OTHER ________________(SPECIFY)

POPULATION COUNCIL
Ideas. Evidence. Impact.

Coronavirus Knowledge Attitudes and Practices

dkjkuk ok;jl tkudkjh] jo;k vkj vH;kl

Consent:

नमस्ते, मेरा नाम .... है। मैं पॉपुलेशन कौंसिल के लिये काम करती/ता हूं। यदि आपको याद हो तो पॉपुलेशन कौंसिल की रिसर्च टीम ने कुछ साल पहले आपका या आपके परिवार के किशोर लड़के/ लड़कियां साक्षात्कार किया था । अब हम कोराना वायरस के बारे में कुछ सामान्य प्रश्न पूछना चाहेंगे जिसमें 20 से 25 मिनट लग सकते हैं, आप इसके बारे में क्या जानते हैं और इससे संबधित आपको क्या चिंताऐं हो सकती हैं। यहां कोई सही या गलत जवाब नहीं हैं और जो जवाब आप देते हैं उससे आपके घर के लिए कोई प्रत्यक्ष लाभ या जुर्माना नहीं होगा। जो जवाब आप देगे वह हमें इस बारे में बेहतर समझ देंगें कि आपके समुदाय के लोग क्या जानते हैं ताकि सरकार और अन्य सहयोगी बेहतर कार्यवाही कर सके। यदि आप सहमत हों, तो आपका दोबारा साक्षात्कार करें।

My name is ______. I am working for Population Council. We interviewed in you or adolescent from your household few years back. Now, we would like to ask some general questions about the Corona virus which may take 20 to 25 minutes, what you know about it and what your concerns might be. There are no right or wrong answers and the answers you provide will not lead to any direct benefits or penalties for your household. The answers that you give, provide us a better understanding of what people in your community know so that the government and other partners can better respond. Can we talk to you?

	**Question and Filters**	**Code/Response Categories**	**Skip To**
q100a	प्रतिभागी है...<br>Respondent is…	किशोर लड़के/लड़कियां/<br>Adolescent/ young person (UDAYA cohort member) ……………1<br>घर का मुखिया/<br>Head of household…………………………………………………..2<br>घर के वयस्क सदस्य (मुखिया नहीं)/<br>Adult member of household (but not head)……………………….3	
q100	उत्तरदाता पुरूष है या महिला?<br>Sex of respondent	पुरूष/ Male..1<br>महिला/ Female..2	
q101	आपकी उम्र कितनी है?<br>How old are you?	उम्र पूर्ण वर्षों में/<br>AGE IN COMPLETED YEARS [ ][ ]	
	अब हम आपसे कोरोना के बारे में कुछ प्रश्न पूछना चाहेंगे।<br>Now we would like to ask you some questions about Corona.		
q102	क्या आपने कभी कोविड–19, कोरोना वायरस या कोरोना नाम की बीमारी के बारे में सुना है?<br>Have you ever heard of a disease called COVID-19, Coronavirus or Corona?	हां/ YES…………………………………………..........…1<br>नहीं/ NO…………………………….......…….........…2<br>कोई जवाब नहीं/ No response…………..…………8	125 (2, 8)
q105	क्या आप मुझे बता सकते हैं कि आप कोरोना के लक्षणों के बारे में क्या जानते हैं?<br>Can you tell me what you know about the symptoms of Corona?<br><br>निर्देशः प्रोब करें – और कुछ?<br>Note: Probe - anything else?<br><br>निर्देशः यदि जवाब बीमार होना या फ्लू है, तो लक्षण बताने के लिए कहें<br>Note: if response sick or flu, ask to specify symptoms	कोराना वायरस के किसी भी लक्षण को नहीं जानते हैं/ No symptoms of Coronavirus known……………………..A<br>बुखार/100.4 डिग्री से उपर तीव्र बुखार/<br>Fever/High Fever above 100.4 degree…………………………B<br>सिरदर्द/ Headache……………………………………………C<br>सूखी खांसी/ Dry cough……………………………………..D<br>डायरिया/ अतिसार/ दस्त/ Diarrhea………………E<br>सांस लेने में परेशानी/Difficulty breathing……………….F<br>खाने की किसी भी वस्तु का स्वाद पता ना लगना/<br>Loss of taste…………………………………………………......G<br>सूंघने की क्षमता में कमी आना/<br>Loss of smell……………………………………………………H<br>थकान/ Tiredness……………………………………………….I	

	बताये गये सभी दर्ज करें<br>Record all mentioned	सीने में दर्द / Chest pain………………………………………J<br>ठंड लगना / Chills……………………………………………K<br>त्वचा पर चकत्ते होना / Rash……………………………L<br>चक्कर आना / Dizziness…………………………………….M<br>अन्य (स्पष्ट करें) /<br>OTHER (SPECIFY) _______________________________X<br>कोई जवाब नहीं / No response……………………………Z	
q106	क्या आप मुझे बता सकते हैं आप कोरोना से बचने के बारे में क्या जानते हैं?<br>Can you tell me what you know about how to prevent Corona?<br><br>निर्देशः प्रोब करें – और कुछ?<br>Note: Probe - anything else?<br><br>बताये गये सभी दर्ज करें<br>Record all mentioned	कोराना वायरस से बचनें के बारे मे नही जानते हैं /<br>No Coronavirus prevention method…………………………...A<br>हाथों को धोना / हैंड सेनिटाइजर इस्तेमाल करना /<br>Wash hands/use hand sanitizer…………………………………B<br>चेहरे को ना छूना / Do not touch face……………………C<br>किसी से हाथ ना मिलाना /<br>Do not shake hands…………………………………………….D<br>घर के बाहर किसी भी वस्तु का ना छूना /<br>Do not touch anything outside of household……………………E<br>लोगों से 2 मीटर की दूरी पर रहें /<br>Stand 2 meters away from people…………………………….F<br>सतहों को रगड़ें / साफ रखें /<br>Scrub/clean surfaces…………………………………………...G<br>घर पर ही रहें जब तक कि अत्यावश्यकता ना हो /<br>Stay home unless urgent………………………………………H<br>चर्च / मस्जिद / मंदिर आदि धार्मिक स्थालों पर ना जायें / Don't go to church/mosque/temple etc………………..I<br>विवाह / अंतिम संस्कार समारोह में ना जायें /<br>Don't go to weddings/funerals………………………………….J<br>डिजीटल मनी का इस्तेमाल करें /<br>Use digital money……………………………………………….K<br>मास्क पहनें / Wear masks……………………………….L<br>देशी शराब पीना / Drink local alcohol…………………...M<br>अन्य लोगों से संपर्क बनाने से पूरी तरह बचना /<br>Avoid contact with other people completely………………….N<br>जिन लोगों के मैं संपर्क में आता हूं उनकी संख्या कम करना /<br>Reduce the number of people I come in contact with……….O<br>क्लोरोक्विन / मलेरिया की दवा लेना /<br>Take chloroquine/malaria medication…………………………P<br>अस्पताल / चिकित्सालय जाने से बचना /<br>Avoid hospitals/clinics…………………………………………..Q<br>सार्वजनिक परिवहन इस्तेमाल करने से बचना /<br>Avoid public transport…………………………………………..R<br>लोगों से 1 मीटर की दूरी पर रहें /<br>Stand 1 meter away from people………………………………S<br>अन्य (स्पष्ट करें) /<br>OTHER (SPECIFY) _______________________________X<br>कोई जवाब नहीं / No response……………………………Z	
q107	क्या आपको लगता है कि आपको कोरोना से संक्रमित होने की संभावना कम है, मध्यम है या अधिक है, या क्या आपको बिलकुल भी जोखिम नहीं है?<br>Do you think your chance of getting infected with Corona is low, medium, or high, or do you have no risk at all?	कम / Low……………………………………………………1<br>मध्यम / Medium……………………………………2<br>अधिक / High……………………………………………3<br>कोई जोखिम नहीं / No risk………………………4<br>पहले से ही कोरोना वायरस है /<br>Already had Coronavirus……………………………5<br>पता नहीं, कोई जवाब नहीं /<br>Don't know, no response…………………………8	109 (2, 3)<br><br>109 (5, 8)

	Question and Filters	Code/Response Categories	Skip To
q108	आपको क्यों लगता है कि आपको कम जोखिम है/जोखिम नहीं है?<br>Why do you think you are at low/not at risk?<br><br>निर्देशः प्रोब करें – और कुछ?<br>Note: Probe - anything else?<br><br>बताये गये सभी दर्ज करें<br>Record all mentioned	मैं जवान/स्वस्थ हूं/ I'm young and healthy……………..A<br>मेरी रक्षा भगवान करता है/ God protects me…………..B<br>गर्म मौसम/वातावरण/ The hot weather/climate………..C<br>मैं अपने गांव/पड़ोस से बाहर यात्रा नहीं की है/<br>I haven't travelled outside my village……………………………..D<br>मैंने अपने जिले/राज्य/देश से बाहर यात्रा नहीं की है/ I haven't travelled outside my district/state/country………..E<br>मेरे गांव/पड़ोस में कोरोना का कोई पौजिटिव केस नहीं है/ No positive cases in my village/neighbourhood.…...F<br>अन्य (स्पष्ट करें)/<br>OTHER (SPECIFY) ________________________________X<br>कोई खास कारण नहीं था/<br>No specific reason………………………………………..…..Y	
q109	क्या आपको लगता है कि आपके गांव/आस–पड़ोस में किसी व्यक्ति को कोरोना से संक्रमित होने की संभावना कम है, मध्यम है या अधिक है, या क्या बिलकुल भी जोखिम नहीं है?<br>Do you think the chance of anyone in your neighbourhood/village getting Coronavirus is low, medium or high, or is there no risk at all?	कम/ Low..1 →<br>मध्यम/ Medium……………………………………2<br>अधिक/ High..3<br>कोई जोखिम नहीं/ No risk……………………….4<br>पता नहीं, कोई जवाब नहीं/<br>Don't know, no response…………..……………….8	118<br><br><br>118
q109a	आपको ऐसा क्यों लगता है कि आपके गांव/ आस–पड़ोस में कोरोना होने की संभावना मध्यम या अधिक है?<br>Why do you think people in your neighbourhood/village are at medium/high risk of getting corona?	लोग भारत के शहरी इलाकों से लौट आए/<br>People returned from urban areas of India………..….A<br>लोग भारत के अन्य राज्यों से लौट आए/<br>People returned from other states of India…………...B<br>विदेश से लोग घर आ गए/<br>People from foreign country came home…………....C<br>हमारे पड़ोस में कोरोना का पौजिटिव केस है/<br>We had positive case in neighbourhood………..……D<br>अन्य (स्पष्ट करें)/<br>OTHER (SPECIFY) ______________________________X<br>कोई खास कारण नहीं था/<br>No specific reason………………….…………………..Y	
q118	आप या आपके घर के सदस्य आजकल कोरोना वायरस से बचाव के लिए क्या–क्या कर रहे है?<br>What have you and your household members been doing to avoid Corona virus?	घर पर ज्यादा समय तक रहे/<br>Stayed at home (not stepping outside)…………………………..A<br>सामाजिक समारोहों में भाग लेना बंद कर दिया (जैसे विवाह/अंतिम संस्कार/चर्च/मस्जिद/मंदिर)/<br>Stopped attending social gatherings (e.g. weddings/funerals/church /mosque/temple)……………………..B<br>स्कूल में या काम पर जाना बंद कर दिया/<br>Stopped attending school or work……………………………….C<br>कम से कम 2 मीटर की दूरी बनाये रखी/<br>Kept a distance of at least 2 meters……………………….……D<br>लोगों को बीमारी के लक्षणों के बारे में सूचित किया/ Informed people of illness symptoms…………….……E<br>ज्यादा बार हाथों को धोया हैंड सेनिटाइजर का इस्तेमाल किया/<br>Washed hands/used hand sanitizer more frequently………….F<br>कोरोना हेल्पलाइन पर संपर्क किया/<br>Contacted Corona helpline………………………………………..G<br>मास्क का उपयोग शुरू कर दिया/<br>Started using masks………………………………………………H<br>दस्ताने का उपयोग शुरू कर दिया/	

		Started using gloves……………………………………………I<br>कुछ भी हीं / Nothing…………………………………………Y<br>कोई जवाब नहीं / No response……………………………Z	
q120	यदि आवश्यकता पड़े तो क्या आपके घर में कोई अलग कमरा या स्थान है ताकि कोई भी अपने आप को दूसरों से अलग करके रह सके?<br>Do you have a separate room or space to practice self-quarantine, if needed?	हां / YES…………………………………………1<br>नहीं / NO…………………………………………2	
q124a	क्या आपकी जान पहचान मे कोई है जो कोरोना वायरस से संक्रमित / पौजिटिव है?<br>Has anyone you are acquainted with been tested positive for coronavirus?	हां / YES…………………………………………1<br>नहीं / NO…………………………………………2	
q125	आपने कितनी कक्षा तक उच्चतम शिक्षा <u>सफलतापूर्वक प्राप्त</u> की है (शिक्षा का स्तर वर्षों में लिखें)<br>What is the highest level of schooling you have <u>successfully completed</u> (in number of years of schooling)?	शिक्षा का स्तर वर्षों में / COMPLETED YEARS OF SCHOOLING [ ][ ]<br>कभी स्कूल नहीं गई है / NEVER ATTENDED SCHOOL……………………00	
q127a	आपके घर में कितने कमरे हैं?<br>How many rooms are there in your household?	कमरों की संख्या NUMBER OF ROOMS [ ][ ]	
q127	आपको मिलाकर, कुल कितने लोग पिछली रात आपके घर में सोये हैं?<br>Including yourself, what is the total number of people who slept in your house last night?	संख्या (0-20) / Number (1-20) [ ][ ]<br>केवल मैं स्वयं / Only myself………………………0	
	आगे पूछे जाने वाले प्रश्न आजकल की परिस्थिति के बारे में हैं जो घर में हो सकती हैं।<br>The following questions are about current situation. You will not be receiving any benefit based on your answers, so we ask you to be as honest as possible.		
n100A	क्या आपके परिवार का कोई सदस्य जिले के बाहर रह रहा है / काम कर रहा है?<br>Has any of your family member living/working outside the district?	मैं स्वयं / Myself……………………………A<br>परिवार का अन्य सदस्य / Other member in my family……………………B<br>कोई नहीं / None……………………………Y →	100
n100B	क्या कोरोना या लॉकडाउन के कारण आपके परिवार के सदस्य जो बाहर रहते थे वह घर वापस आ गएं ?<br>Did the family member came back due to Corona or lockdown?	हां / YES…………………………………………1<br>नहीं / NO…………………………………………2	
n100C	क्या आपके परिवार का कोई सदस्य इस लॉकडाउन के कारण कही फंस गया है?<br>Do you have any family member stranded elsewhere because of the lockdown?	हां / YES…………………………………………1<br>नहीं / NO…………………………………………2	
n100	लॉकडाउन के कारण आपकी या आपके परिवार के किसी सदस्य नौकरी छूट गई है या आमदनी या आजीविका का स्त्रोत समाप्त हो गया है?<br>Have you or any member of your family lost your job, income source or livelihood due to the lockdown?	हां / YES…………………………………………1<br>नहीं / NO…………………………………………2 }<br>पहले से ही बेरोजगार हैं / Previously unemployed……………………………3 }	104

	Question and Filters	Code/Response Categories	Skip To
n101	आपका या परिवार के सदस्य जिनकी नौकरी छूट गई है का क्या व्यवसाय/नौकरी था?<br>What is your occupation or the occupation of the family member who lost their job/income?<br><br>व्यवसाय स्पष्ट करें–<br>Specify Occupation_______________	अनौपचारिक सेक्टर/ Informal sector……………………..A<br>सरकारी कर्मचारी/ Government employee……………....B<br>फार्मेसी कर्मचारी/ Pharmacy worker……………………..C<br>स्वास्थ्य देखभालकर्मी/ Healthcare worker……………..D<br>दुकान मालिक/ Shop owner…………………………….E<br>गृहणी/ Homemaker……………………………………….F<br>निजी सेक्टर /Private sector……………………………..G<br>अन्य (स्पष्ट करें)/<br>OTHER (SPECIFY) ……………………………………….X<br>कोई नहीं, कोई जवाब नहीं/<br>None, no response……………………………………….Y	
n101A	आप या आपके परिवार के सदस्य कहां थे जब नौकरी/पैसा कमाने का साधन/नौकरी छूट गई?<br>Where were you or your family member who lost job/income sources working?	जिला में ही/Within District……………………….A<br>जिला से बाहर/Outside District…………………..B	
n104	इस घर में रहने वाले कुल कितने लोग हैं जिनकी उम्र 60 साल से अधिक है?<br>What is the total number of people who reside or live in this house who are older than 60?	संख्या (0-10) /<br>Number (0-10) [ ][ ]	
n107	क्या आपको या आपके परिवार के सदस्यों में किसी को इनमें से कोई बीमारियां या रोग हैं?<br>Do you or any other family member have any of the conditions or diseases?	हां/ Yes — नहीं/ No	
	छमा/ Asthma	1 — 2	
	मधुमेह (शुगर)/ Diabetes	1 — 2	
	मोटापा (सामान्य से अधिक वजन होना)/ Obesity	1 — 2	
	किडनी/गुर्दा की बीमारी/ Kidney disease	1 — 2	
	दिल की बीमारी/ Heart disease	1 — 2	
n108	आपके घर के वर्तमान आर्थिक संसाधनों के साथ, आपका परिवार कितने दिनों तक जीवित रह सकता है?<br>With your current household financial resources, how long could your family survive?	एक हफ्ते से कम/<br>Less than one week……………………………………………1<br>एक हफ्ता से दो हफ्ते/<br>One week to two weeks…………………………………………2<br>दो हफ्ते से एक महीना/<br>Two weeks to one month…………………………………….3<br>एक महीना से ज्यादा/<br>More than one month…………………………………………..4	
n109	(महिला उत्तरदाता के लिए): लॉकडॉउन के दौरान पिछले 15 दिनों में, क्या आपको घर में किसी भी प्रकार की हिंसा का अनुभव हुआ है?<br>[For female respondents]: Under lockdown, have you experienced any violence in the home in the last 15 days?	हां/YES…………………………………………...........1<br>नहीं/NO……………………………........................2<br>पता नहीं, कोई जवाब नहीं/<br>Don't know, no response……………………………8	(2, 8) N110A

	Question and Filters	Code/Response Categories	Skip To
N109A	(महिला उत्तरदाता के लिए): लॉकडॉउन के दौरान, पिछले 15 दिनों में, क्या आपने हिंसा को बढ़ते हुए देखा है?<br>[For female respondents]: Under lockdown, have you observed any increase in violence in the last 15 days?	हां / YES……………………………………..........1<br>नहीं / NO………………………….......…......…2	
n110A	(महिला उत्तरदाता के लिए): लॉकडाउन के दौरान, क्या आपको नीचे दिए प्रजनन स्वास्थ्य सेवाएं में से कोई सेवा मिल रही है?<br>[for female respondents]: Under lockdown, are you getting any of the following reproductive health services?<br>निर्देश: प्रत्येक प्रश्न पूछें<br>Note: Ask each question.	हां / Yes — नहीं / No — जरुरत नहीं / Not required<br>1. प्रसवपूर्व देखभाल Antenatal care 1 2 3<br>2. परिवार नियोजन Family planning 1 2 3<br>3. बच्चे का टीकाकरण Child immunization 1 2 3<br>4. पोषण सेवाएं Nutrition services 1 2 3	
n111	सरकार द्वारा किये गये लॉकडाउन अवधि के दौरान आपकी/आपके घर की महत्वपूर्ण जरूरतें क्या हैं?<br>Given that our country is under lockdown, what are your/your household critical needs to go through this phase?	भोजन (सब्जी, दूध की वस्तुओं सहित सभी राशन आइटम शामिल हैं) / Food (include all ration items, including vegetable, milk items)...A<br>पानी / Water..B<br>दवाऐं / Medicine………………………………………………C<br>फोन पर टौकटाइम/ डाटा पैक/ Talk time/data bundles……………………………………………..D<br>पैसा / Money...E<br>रसोई गैस या खाना पकाने का ईंधन/ LPG or cooking fuels……………………………………………F.<br>अन्य/ Other___________________________________X	
n112	लॉकडाउन के दौरान, क्या आप अपने आप को अकेला, उदास या चिड़चिड़े महसूस करते हैं?<br>Under the lockdown, do you feel lonely, depressed or irritable?	कभी नहीं/ Never……………………………………1<br>कभी–कभी/ Sometimes………………………….2<br>ज्यादातर समय/ Most of the time……………….3	
Q133	क्या हमे भविष्य में आपसे दोबारा संपर्क करने के लिए आपकी अनुमति है?<br>Do we have your permission to recontact you in the future?	हां / YES…………………………………...….1<br>नहीं / NO……………………………......…....2	

आपके समय के लिए शुक्रिया। यदि हमें किसी अतिरिक्त जानकारी की आवश्यकता है तो हम आपके संपर्क में रहेंगे। कृपया घर पर रहें, और अक्सर अपने हाथों को पानी और साबुन से धोएं। हमारी शुभकामनाएं आपके साथ हैं।

Thank you for your time. We will be in touch with you if we need any additional information. Please continue to practice physical distancing, stay home, and frequently wash your hands with water and soap. Our best wishes are with you.
