# Supplementary material for: Gender specific differences in COVID-19 knowledge, behavior and health effects among adolescents and young adults in Uttar Pradesh and Bihar, India
Source: PLoS One. 2020 Dec 17;15(12):e0244053. doi: 10.1371/journal.pone.0244053 (PMC7746145; doi:10.1371/journal.pone.0244053)
Supplement: Attachment — Submitted filename: response to reviewers.docx [file pone.0244053.s003.docx]

Journal Requirements:

When submitting your revision, we need you to address these additional requirements.

 1. Please ensure that your manuscript meets PLOS ONE's style requirements, including those for file naming. The PLOS ONE style templates can be found at

<https://journals.plos.org/plosone/s/file?id=wjVg/PLOSOne_formatting_sample_main_body.pdf> and

<https://journals.plos.org/plosone/s/file?id=ba62/PLOSOne_formatting_sample_title_authors_affiliations.pdf>

 2. Please include additional information regarding the survey or questionnaire used in the study and ensure that you have provided sufficient details that others could replicate the analyses.

For instance, the content development and validation.

If the questionnaire it is not under a copyright more restrictive than CC-BY, please include a copy, in both the original language and English, as Supporting Information.

The questionnaire is freely available through the Harvard Dataverse, here: <https://dataverse.harvard.edu/dataset.xhtml?persistentId=doi:10.7910/DVN/8ZVOKW>

We have also added it as supporting information.

3. We note that you have stated that you will provide repository information for your data at acceptance. Should your manuscript be accepted for publication, we will hold it until you provide the relevant accession numbers or DOIs necessary to access your data. If you wish to make changes to your Data Availability statement, please describe these changes in your cover letter and we will update your Data Availability statement to reflect the information you provide.

The dataset is available upon request through the Harvard Dataverse, here is the link: <https://dataverse.harvard.edu/dataset.xhtml?persistentId=doi:10.7910/DVN/8ZVOKW>

When someone accesses this page, they can clink “access dataset” to receive it.

[Note: HTML markup is below. Please do not edit.]


5. Review Comments to the Author

Please use the space provided to explain your answers to the questions above. You may also include additional comments for the author, including concerns about dual publication, research ethics, or publication ethics. (Please upload your review as an attachment if it exceeds 20,000 characters)

**Reviewer #1:** This manuscript highlights the gender differences in knowledge and adoption of preventive practices against COVID-19 in two provinces in India, as well as mental health consequences that are particularly pronounced among women. The discussions contained in this paper are very important to highlight the necessity of COVID-19 response guidance with adequate gender considerations. This is especially relevant in the context of India where the cultural influence over gender norms is strong and as a country where the spread of the virus has been particularly severe. However, parts of the paper will need to be restructured in order to improve the clarity and flow of the manuscript as a narrative. In particular, it is important that challenges to health care access for women is properly conceptualized in the introduction as a study aim in order to justify its subsequent analysis. Some discrepancies and inconsistencies in language, though minor issues, are important to resolve (namely, the use of women/men vs. female/male and using a term that is broader than “depression” due to the simplistic measure).

We include more detail below in the specific responses. Overall, thank you for these useful comments. We have taken steps to restructure the background and discussion sections for clarity, add details regarding health access for women, and to resolve inconsistencies in language (specifically, using women/men to denote gender and the term ‘depressive symptoms’ as our measure is very simple).

Below are more detailed comments and recommendations:

Abstract
1. It is not immediately clear why it is highlighted that knowledge was the only predictor of practice of preventive behaviors among women, especially since we see later that it is a significant predictor for men as well. As an important key finding, the authors may consider further expanding on the implications of this key finding, e.g. that because knowledge is the only predictor for females, education programs/information campaigns are particularly important as an intervention with gender considerations.

We have edited the abstract to reflect this suggestion regarding knowledge.

2. Great, succinct summary of key findings in lines 39-41.

Thank you!

Introduction
3. Could the authors rephrase, or be more specific about what they mean by “the negative secondary effects of crises” (line 56)? Although examples of what these secondary effects include are described in subsequent paragraphs of the introduction, without an upfront definition or description, it is not immediately clear to readers what this means, or why the authors are focusing on said secondary effects as opposed to the “primary effect” of infection.

While research on COVID-19 infections is critical, testing is not widespread or available for analysis. Additionally, our team at the time of data collection was constrained by requirements to only collect information on the phone. Instead of focusing on the effect of infection with coronavirus, this paper instead focuses on the downstream (“secondary”) effects of the pandemic – specifically economic insecurity and utilization of essential health services. We have clarified in the introduction and removed the term ‘secondary’.

4. What do the authors mean by “structural facilitators” (line 71)?

We have added a sentence at line 77 to clarify. What we refer to here is structural factors such as policies or physical infrastructure that would prevent adoption of healthy behaviors, – e.g., promoting handwashing with soap only works if soap and water are available.

5. The paragraph starting on line 73 needs to be restructured to have one main idea. Currently, I can identify four separate ideas: (1) There are a number of negative repercussions as a result of COVID-19 pandemic, particularly barriers to accessing health care, (2) inability to access health care is particularly problematic for women, (3) the prevalence of gender-based violence and adverse mental health outcomes may increase during lockdowns, (4) depression in response to COVID-19 has been reported among adolescents. The background information provided to readers should be focused and tailored to the authors’ stated research aims in line 87: to highlight “the gender specific variation in COVID-19 knowledge and practice of preventive behaviors, and mental health effects”.

We have split these into two paragraphs to add additional information and references.

6. If one of the study aims was to examine the adolescent and young adult population in particular, an additional paragraph justifying why is warranted. However, it could also be the case that the primary aim of the study was to examine gender differences, but using a study sample of adolescents and young adults (potentially due to reasons of feasibility / harnessing an existing cohort study), in which case this should just be acknowledged.

We have clarified in the introduction and in the methods that this is the second case – we surveyed adolescent and young adults sampled from the UDAYA project, the aim of the paper is to explore gender differences related to COVID-19.

7. The authors may consider removing two sentences in lines 88 to 91, as it is information repeated from earlier in the introduction.
We have removed these lines, thank you for catching this repetition.

Methods
8. In line 101, the authors should specify that the original study objectives of the UDAYA study were about acquisition of assets and transition into adulthood, so it is clear that it is distinct from the objectives of the present study.

We have clarified that the study objectives originally related to assets and transition to adulthood, and that we sampled from this existing cohort.

9. It is good to see that the authors explicitly state that while the survey collected information on the participants’ sex, the study would adopt the lens of gender. However, the language of gender (women/men) should then be used consistently throughout the paper.

We have consistently switched male/female to refer to women/men throughout the paper and added the following to clarify this language at line 158: “ In the survey participants self-reported their sex as male or female; throughout this paper we will refer to respondents as men and women to illustrate that our analysis reports how the pandemic impacts gender (the socially constructed characteristics of men and women) not biological sex.”

10. The sentence “All survey responses were tabulated by gender…” in lines 140-141 should be moved to last paragraph of the methods where the statistical analysis approaches are described.

We have moved this sentence to be where the statistical analysis is described.

11. I do not think the reporting “feeling lonely, depressed or irritable” can be accurately labelled as experiencing depression. I suggest the authors consider using an alternate term that is broader, such as “adverse mental health effect” (as in the abstract). This comment also applies to all sections of the paper, including figures and tables.

Thank you for this comment. We agree that these self-reported symptoms cannot constitute a depression diagnosis. We have clarified that these are “depressive symptoms” rather than a diagnosis of depression. While self-reporting such symptoms could be indicative of more severe outcomes, an otherwise mentally healthy individual could be experiencing these symptoms.

12. It would be helpful if the word “caste” (line 150) were defined for the international reader.

We agree these terms are important to define for an international reader, caste and OBC are defined at line 186-188.

13. Just as the other variables are described, the operationalization of education should also be included. In particular, an explanation for how years of education have been categorized should be provided, i.e. why is the range for the middle category of 8-9 years of education so narrow, compared to the other two categories?

We have added a sentence at line 180 to clarify the categorization of education attainment into 3 categories. Grade 8 indicates completion of primary school and 10 completion of secondary school, so 8-9 indicates current secondary education enrollment.

14. A similar comment for religion – how were they categorized? Tables 2 and 3 state that Muslim is compared to “Hindu, other religions” whereas Table 1 suggests that the category only refers to Hindu. If other religions were combined with Hindu into one category, this needs to be specified.

Only 9 individuals classify their religion as something other than Hindu or Muslim; we have indicated this in Table 1, and in the models have excluded these 9 to compare Hindu to Muslim participants. This is also clarified in the methods section where variables are defined in line 184-185.

15. In line 158, the authors state that logistic regression models were used, although it seems like linear probability models were used throughout the paper.

Thank you for catching this, we had initially used logistic regression then switched to linear probability models for ease of interpretation – we have deleted this.

16. The authors may also consider clarifying that three models were constructed for each of the three outcomes of interest.

We have clarified that we ran 3 models in lines 196.

17. It is unclear what is meant by “Models were constructed to explore characteristics associated with reporting household employment lost due to COVID-19”, as loss of employment was not described as one of the outcome variables.

Thank you for catching this as well, a previous version had explored this variable but it has been removed. We have deleted the sentence.

Results
18. I would suggest avoiding the language of “more likely” or “less likely” to compare the survey responses of women vs. men when they are expressed in percentages, and to reserve this for reporting probabilities. Instead, for example, the authors may simply report that fewer women (40%) were aware of the main symptoms of COVID-19, compared to men (53%).

We have revised accordingly.

19. The phrase “among male respondents in the male only model” (line 209) can be simplified to avoid repetition.

We have simplified this sentence to avoid repetition.

20. The findings about health service access (from line 220) come as a bit of a surprise to the reader, as this is not stated as a study aim, nor is this step described in the methods. If exploring challenges in accessing healthcare among women is an additional study objective, this needs to be reflected in the introduction and methods, with clear justifications for why this is a gendered issue.

We have added more detail about health service access to the background.

Discussion
21. It may be more accurate to rephrase the finding in lines 232 and 239 (“Overall, female respondents had less accurate information of COVID-19 symptoms…”) to instead say that women were less likely to be able to identify all three COVID-19 symptoms correctly. It can then be postulated that this may be because they had less access to accurate knowledge of COVID-19, compared to men.

We have edited this sentence.

22. The way the sentence “This is also supported by a recent study that found differential COVID-19 risk and mortality by gender and age, suggesting most infections are among men because males are more likely to leave home for work and for socializing due to gendered social norms” (line 249-252) is structured may confuse readers, as the previous sentence was talking about knowledge of viruses, whereas the focus of this sentence jumps to risk of infection. I suggest restructuring the sentence to emphasize the similar role of gendered social norms in affecting both knowledge and infection risk.

We have edited this section for clarity.

23. It would also be beneficial to mention that this second study was also conducted in India.

Yes! Edited.

24. Similar to my above comment for the abstract, authors should expand on the significance of knowledge being the only characteristic associated with adoption of behaviors among women, compared to men where sociodemographic variables also had a role to play.

We have added a sentence at line 311 to highlight that knowledge was the only factor associated with adoption of behaviors among women, suggesting other unmeasured characteristics may be involved or causing the variation in adoption of promoted behaviors.

25. Findings about access to health services (line 274) should be a different paragraph, as it is a separate finding from the mental health outcomes.

We have separated this into two paragraphs for clarity.

26. What do the authors mean by “both sets of questions were spontaneous responses only” (line 293)?
We have edited this line for clarity. We meant that although we are slightly concerned that having the participant respond to behaviors they were aware of before answering behaviors they were doing may have served as a reminder, this should be minimal since the questions were spontaneously given by the respondent, not listed out by the interviewer/

27. The limitation of the mental health measure is important and well-described.
Thank you! It is challenging with a phone survey but we thought this dimension of health important to capture.

Tables
28. (Table 1) Could the authors clarify what ‘Total’ in the top left corner refers to?

Deleted, this should not be on the left side.

29. (Table 1) Why are the responses to only two of four preventive measures presented in particular?

We have deleted these to focus on the overall variable for all 4 main preventive behaviors.

30. (Table 1) Antenatal care should be written out in full since the abbreviation “ANC” has not been defined elsewhere in the manuscript.

We have spelled out ANC in the table and discussion.

31. (Tables 2 and 3) The reference category of ‘sex of the respondent’ should be specified.
Thank you for catching this! We have edited.

32. (Tables 2 and 3) The abbreviation “UP” for Uttar Pradesh should be defined earlier in the manuscript.
Thank you, we have added this abbreviation earlier.

33. (Tables 2, 3 and 4) Whether “NA” and “REF” are used should be consistent.

Thank you, we use NA to denote that the row is not applicable (e.g., there is no gender men v women variable in the men only or women only model) and REF to denote the reference group if more than 2 categories.

**Reviewer #2:** This area of investigation is not novel nor unique. There is a clear bias in the number of participants; more females were included in the survey. The statistical analysis section was not included in the methodology. The results were presented accurately, but the discussion section again was week and did not include comparison with findings from similar studies in the region. The study has a number of limitation which could be prevented with better planning, beside the disadvantages of using this type of phone surveys.

Thank you for these comments. While we agree this analysis has some limitations, overall we feel that this is still an important piece of work given how early in the pandemic it was initiated and feel it is unique in presenting the experiences of adolescents early into the pandemic and associated ‘lockdown’. Specifically:

1) While the gender difference on the impact of COVID-19 are not unique or novel, our study is one of the first systematic, quantitative studies of how COVID-19 is impacting the lives of adolescent and young adults in Bihar and UP. Very little research on COVID-19 is exploring the effects on adolescents and young adults, specifically, as mortality rates are higher among older adults. However, the economic and social effects will be felt by all segments of the population, in different ways. A strength of our study is that we were able to sample from an underlying cohort, instead of using random digit dialing or other techniques that are even less representative. As noted, we were also limited by the use of phone surveys – however, these were the only safe approach to data collection early on during the pandemic. Given these challenges, we feel that the paper represents an important understanding of how the pandemic has already been affecting and shaping the lives of households in two regions in India.
2) We agree that the respondents are skewed by gender and have added a sentence to the limitations to note this.
3) The statistical analysis section in the methodology is at the end of the “data management and analysis” section which may have been missed. The paragraph starts at line 191. We have reorganized this paragraph slightly for clarity.
4) We have added additional references to the discussion section; however, at the time of drafting, very few papers were published regarding COVID-19 dynamics in the region.
5) We agree that phone surveys are not ideal, however, to ensure safety for our interviewers and for participants we were not able to do face-to-face surveys. A strength of our study is that we have met and interviewed these households before, so we had some information on them from their last interview in person, and also they had higher trust in our interviewers potentially increasing their honesty and willingness to participate despite the phone-based nature of our survey. We describe in our limitations section that we understand phone-based surveys are challenging especially when asking about mental health and other sensitive topics.

**Reviewer #3**: 1. The study shows that there is 95% responce rate in the telephonic survey. it may be further be looked into as responce rate seems to be very high (line no. 115).

We note that 51% of phone numbers were no longer functional, but of numbers we did successfully reach only 5% refused to participate.

2. in the line 222, Nutrition services is 51%, it may be the typo error of 52%.

Thank you, we have corrected this typo.

3. The study may not reflect the true representation of the population covered in the selected states. As most of the respondants may mot have the phone/ Mobile no. to interview, It may be considered as the limitation of the study.

This is a great point. We note in the limitations sections that we could only reach a subset of individuals with mobile phones thus making this sample not representative of the larger population.
